# Rupture bilatérale du ligament croisé antérieur chez un joueur de ski traitée par une ligamentoplastie DIDT sous arthroscopie en un seul temps opératoire

**DOI:** 10.11604/pamj.2016.23.37.8324

**Published:** 2016-02-10

**Authors:** Adil El Alaoui, Ilyas Rabhi

**Affiliations:** 1 Service de Chirurgie Orthopédique du Centre Hospitalier de Chambéry, Chambéry, France

**Keywords:** Anteriorcruciate ligament, ligamentoplastie, DIDT, Anterior cruciate ligament, arthroscopic surgery, DIDT

## Image en medicine

Un patient de 25 ans, victime d'un accident de ski (chute les 2 genoux en valgus flexion rotation externe) occasionnant chez lui une entorse grave des 2 genoux. Le patient a bénéficié d'une immobilisation par des genouillères plâtrées et revu à la consultation après 1 mois. Après 12 séances de rééducation on a noté une bonne évolution sur le plan de la douleur, en revanche le patient garde une instabilité avec sensation de dérobement à la marche et un signe de Trillat Lachman positif au niveau des 2 genoux, d'où l'intérêt de réaliser une IRM des genoux qui a confirmé une rupture bilatérale du ligament croisé antérieur (A, B). Nous avons réalisé une ligamentoplastie du LCA par la technique de DIDT au niveau des 2 genoux en un seul temps opératoire en commençant par le côté gauche. Les suites post-opératoires étaient simples, les radiographies de contrôles face et profil des 2 genoux ont montré un bon positionnement des implants (C, D) et une rééducation a été entreprise le lendemain de l'intervention. La reprise de l'activité sportive était sans problème au 6^éme^mois.

**Figure 1:**
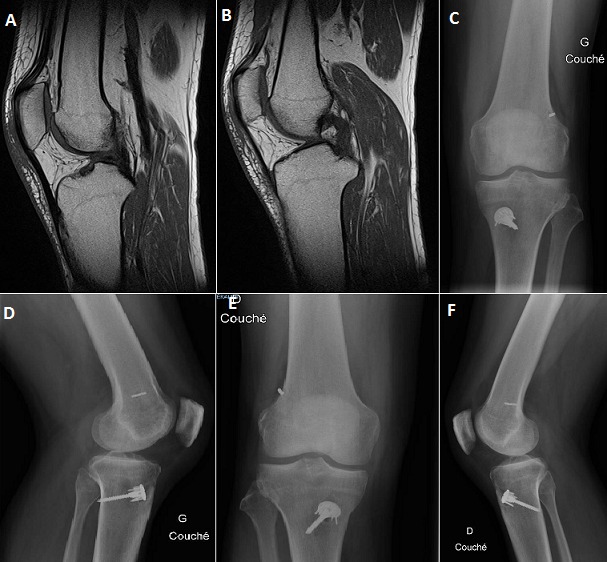
(A) rupture du LCA sur l'IRM du genou gauche; (B): rupture du LCA sur l'IRM du genou droite; (C) bon positionnement du transplant du LCA sur la radiographie de profil du genou gauche; (D) bon positionnement du transplant du LCA sur la radiographie de face du genou droit

